# The Emerging Roles of NDR1/2 in Infection and Inflammation

**DOI:** 10.3389/fimmu.2020.00534

**Published:** 2020-03-24

**Authors:** Xiaolan Ye, Naomi Ong, Huazhang An, Yuejuan Zheng

**Affiliations:** ^1^Center for Traditional Chinese Medicine and Immunology Research, School of Basic Medical Sciences, Shanghai University of Traditional Chinese Medicine, Shanghai, China; ^2^Center for Translational Medicine, Clinical Cancer Institute, Second Military Medical University, Shanghai, China

**Keywords:** NDR1, NDR2, innate immunity, infection, inflammation, Hippo signaling pathway

## Abstract

The nuclear Dbf2-related (NDR) kinases NDR1 and NDR2 belong to the NDR/LATS (large tumor suppressor) subfamily in the Hippo signaling pathway. They are highly conserved from yeast to humans. It is well-known that NDR1/2 control important cellular processes, such as morphological changes, centrosome duplication, cell proliferation, and apoptosis. Recent studies revealed that NDR1/2 also play important roles in the regulation of infection and inflammation. In this review, we summarized the roles of NDR1/2 in the modulation of inflammation induced by cytokines and innate immune response against the infection of bacteria and viruses, emphasizing on how NDR1/2 regulate signaling transduction through Hippo pathway-dependent and -independent manners.

## Introduction

The nuclear Dbf2-related (NDR) kinase NDR1 and NDR2 are also known as serine/threonine kinase 38 (STK38) and serine/threonine kinase 38 like (STK38L), respectively. They are two members of the NDR/LATS kinase family, a subfamily of the AGC (protein kinase A/G/C PKA/PKG/PKC-like) group of serine/threonine kinases, which are highly conserved from yeast to humans (1, 2). The first NDR serine/threonine kinase, Dbf2p, was discovered in budding yeast (3) then followed by the identification of the homologues in human cells (4). The mammalian genome encodes four members of the NDR/LATS kinase family: NDR1 (STK38), NDR2 (STK38L), LATS1 and LATS2 (1). The NDR orthologs are also found in different species: Cbk1p in *Saccharomyces cerevisiae*, Orb6p in *Schizosaccharomyces pombe*, sensory axon guidance-1 (SAX-1) in *Caenorhabditis elegans*, Tricornered (Trc) in *Drosophila melanogaster* (5). NDR1 (Stk38) mainly distributes in the nuclei. NDR2, on the other hand, is defined as a cytoplasmic kinase (4–7). In addition to a central kinase catalytic domain, NDR1 and NDR2 each has a conserved N-terminal regulatory domain (NTR) and a C-terminal hydrophobic motif (8). NDR1/2 have been regarded as protein kinases that are involved in a variety of biological processes, including morphological changes, centrosome duplication, cell cycle and apoptosis (9). Besides, studies also showed that NDR kinases are involved in embryonic development (10), neurodevelopment (11–14), and cancer biology (15, 16). Originally identified in Drosophila, the Hippo pathway is a highly conserved signaling pathway that controls organ size. The core components of the Hippo pathway in mammals include: mammalian STE20-like serine/threonine protein kinases 1 and 2 (MST1/2), the AGC serine/threonine protein kinases large tumor suppressor 1 and 2 (LAST1/2), Salvador family WW domain-containing protein 1 (SAV1), monopolar spindle-one-binder protein 1(MOB1), the transcriptional co-activator Yes-associated protein (YAP) and transcriptional co-activator with PDZ-binding motif (TAZ) (17–22). YAP/TAZ translocate to nuclei and bind to transcription factors, such as TEAD1/2/3/4, to induce the expression of target genes that control cell proliferation, survival, and migration (23–26). When Hippo pathway is activated, MST1/2 phosphorylate SAV1, MOB1, and LATS1/2. After LATS1/2 kinases get activated, they phosphorylate YAP/TAZ. This results in the cytoplasmic sequestration and degradation of YAP/TAZ, which inhibits YAP/TAZ-driven cell proliferation, survival, and migration. NDR1/2 are newly identified as members of the Hippo pathway (5, 18, 27), and were reported to play similar roles like LATS1/2 as upstream kinases of YAP (28–33). Recent studies demonstrated critical functions of the Hippo signaling in innate immunity (17, 24, 34). For example, Mst1/2 knockout mice are more susceptible to cecal ligation and puncture (CLP)-induced sepsis compared to wild-type mice (35). Emerging evidences have revealed that NDR1/2 also play pivotal roles in innate immunity. Here, we reviewed the roles of NDR1/2 in inflammation and antimicrobial innate immune response. We focused on their regulatory roles in innate immunity in Hippo pathway-dependent and -independent manners.

## NDR Regulates Pattern Recognition Receptor-Mediated Innate Immunity

The innate immune system is the first line of host defense against the invasion of microbes, including bacteria, viruses and fungi. Innate immune response is initiated by the recognition of pathogen-associated molecular patterns (PAMPs) of pathogens and damage-associated molecular patterns (DAMPs) of damaged cells by pattern-recognition receptors (PRRs). The members of PRRs include Toll-like receptors (TLRs), C-type lectin receptors (CLRs), retinoic acid-inducible gene (RIG)-I-like receptors (RLRs), NOD-like receptors (NLRs) and DNA sensors. PAMPs are conserved components of pathogens, such as lipopolysaccharide (LPS), mannose, peptidoglycan (PGN), dextran, teichoic acid (LTA), nucleic acids (DNA, RNA), peptide substances (flagella, etc.), lipoproteins, etc. After PAMPs of invading microbes are recognized by PRRs, the downstream signaling pathways of PRRs are activated to elicit innate immune response, accompanied by the secretion of inflammatory cytokines and type I interferons (36, 37). CpG DNA is a typical ligand of TLR9 located on the membrane of endosomes. Our previous research demonstrated that NDR1 (Stk38) is a negative regulator of TLR9-mediated immune response in macrophages. Mechanistically, NDR1 binds with ubiquitin E3 ligase Smurf1. This interaction promotes Smurf1-mediated ubiquitination and degradation of mitogen-activated protein kinase kinase 2 (MEKK2), which is essential for CpG-induced ERK1/2 activation and subsequent production of TNF-α and IL-6. However, MEKK2 is not required for LPS-induced TNF-α and IL-6 production. Consequently, NDR1 inhibits ERK1/2 activation and decreases the production of TNF-α and IL-6 induced by CpG in macrophages. In contrast, NDR1 deficiency only slightly affects LPS-induced cytokine secretion. NDR1 deficiency also increases CpG-induced pro-inflammatory cytokine production *in vivo*. For instance, Stk38-deficient mice infected with *Escherichia coli* had been found to secrete higher levels of TNF-α, IL-6, and show a higher mortality rate than control wild-type mice. Stk38-deficiency also renders mice more susceptible to CLP-induced polymicrobial sepsis than control mice. Similarly, knockdown of NDR2 (Stk38L) with siRNA increased CpG-induced IL-6 secretion, suggesting that NDR2 is functionally similar to NDR1 in regulating the production of TLR9-mediated inflammatory cytokines. Taken together, our results showed that NDR1 prevents the excessive production of inflammatory cytokines by inhibiting TLR9-mediated innate immune response. Thus, NDR1 plays a significant role in protecting the host from TLR9-mediated inflammation (38).

**Figure 1:**
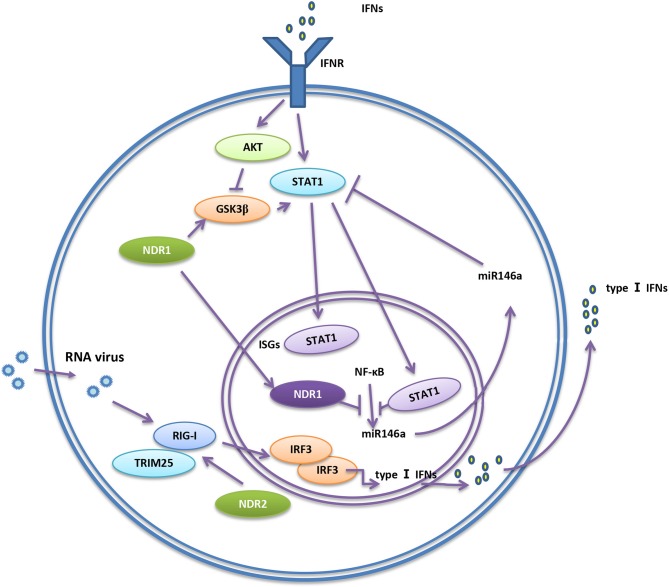
NDR1/2 regulate RIG-I-mediated innate immunity. RIG-I senses virus nucleic acids of viruses and activates downstream signaling pathways to initiate immune response. NDR2 directly associate with RIG-I and TRIM25, thus facilitating the formation of RIG-I/TRIM25 complex and enhancing the polyubiquitination of RIG-I. The ubiquitination of RIG-I further promotes the production of type I IFNs, so the antiviral immune response is enhanced. NDR1 promotes the activity of GSK3β. GSK3β promotes the activation of STAT1, then facilitates the expression of IFN-stimulated genes (ISGs). STAT1 is inhibited by miR146a. Binding to miR146a promoter, NDR1 inhibits NF-κB-mediated miR146a expression, and subsequently releases the inhibition of STAT1 expression by miR146a.

A previous study suggested that NDR1 and NDR2 kinases were incorporated into HIV-1 particles. Furthermore, NDR1 and NDR2 can be cleaved by the HIV-1 protease, which inhibits the activity of NDR1/2 (39). This finding draws our attention to the connection between NDR1/2 and viral infection. MiR146a inhibits TLR signaling by targeting IRAK1, TRAF6, STAT1, and IRAK2 (40–44), which are important for antiviral immune response. A recent study showed that NDR1 acts as a transcriptional regulator by binding to the intergenic region of miR146a, which dampens miR146a transcription to promote the translation of STAT1. This takes place independently of the NDR kinase activity. STAT1 translation subsequently increases the production of type I IFN, pro-inflammatory cytokines and interferon-stimulated genes (ISGs) for the antiviral immune response. These findings revealed that NDR1 positively regulates type I and type II IFN pathways and enhances antiviral immune response (6). Glycogen synthase kinase 3β (GSK3β) and STAT1 are important participants in the antiviral immune response. GSK3β promotes IFN-induced STAT1 activation (45–47). While GSK3β inhibits NDR1 activation, NDR1 decreases the phosphorylation of GSK3β, promotes GSK3β activation and facilitates the production of type I IFNs induced by poly (I:C) (7, 48). Meanwhile, NDR2 was reported to promote RIG-I-mediated antiviral immune response by directly associating with RIG-I and TRIM25, thus facilitating the forming of RIG-I/TRIM25 complex and enhancing K63-linked polyubiquitination of RIG-I (49) (Figure 1). Overall, these findings demonstrated that NDR1/2 down-regulates TLR-mediated inflammation but positively regulates RIG-I-mediated antiviral immune response. It is unclear why NDR1 inhibits CpG-induced inflammatory cytokine production but increases virus-induced inflammatory cytokine production. It is possible that the target of NDR1, MEKK2, which promotes CpG-induced inflammatory cytokine production, plays a different role in antiviral innate immunity. As reported, MEKK2 in tumor-derived exosomes antagonizes innate antiviral immunity (50). In addition, CpG triggers TLR signal transduction and inflammatory cytokine production much more rapid than virus infection. It can't be ruled out that alteration of miR146a and STAT1 by NDR1 are not as efficient in upregulating CpG-induced inflammatory cytokine production as in upregulating virus-induced inflammatory cytokine production. The negative or positive role of NDR1 in CpG and virus induced innate immunity might be the net results of its regulation of MEKK2, STAT1, GSK3, and other unknown molecules under different conditions.

## NDR Regulates Cytokine-Induced Inflammation

**Figure 2:**
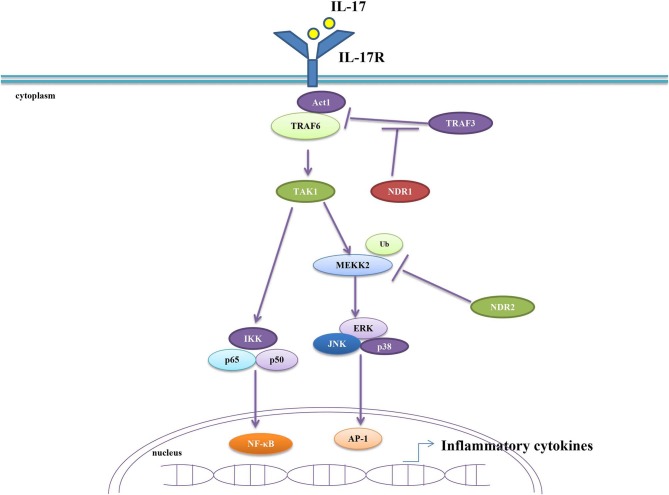
NDR1/2 regulate IL-17-induced inflammatory response. NDR1 competitively binds with TRAF3 and consequently dampens TRAF3-inhibited combination between Act1and TRAF6. This results in the enhanced IL-17 signaling and the increased production of inflammatory cytokines. NDR2 promotes the ubiquitination and degradation of MEKK2 to inhibit IL-17 signaling, thus preventing the excessive secretion of inflammatory cytokines.

Infection and tissue injury are the two main causes of inflammation. In these circumstances, the immune system releases pro-inflammatory cytokines to eliminate pathogens or damaged cells and releases anti-inflammatory cytokines to balance inflammatory response, preventing immune injury. Both previous cytokines form the delicate balance of the immune system. Excessive secretion of pro-inflammatory cytokines can cause serious inflammatory diseases (51). Emerging evidences uncovered that major inflammatory cytokines tumor necrosis factor alpha (TNF-α) and interleukin 17 (IL-17) are associated with autoimmune diseases (52–55). Specifically, IL-17 participates in encephalomyelitis (EAE), rheumatoid arthritis (RA) and IBD. Moreover, IL-17 levels were found to be elevated in patients with multiple sclerosis (MS) and ulcerative colitis (UC) (56–60). NDR1 promotes TNFα-induced NF-κB activation via its kinase activity by interacting with multiple signal components in NF-κB signaling pathway. Thus, it acts as a positive regulator in TNFα-induced inflammation (61). A study from Ma C demonstrated that NDR1 promotes the pathological process of IBD and EAE *in vivo* by facilitating IL-17-mediated and TNF-α-mediated inflammation. NDR1 competitively binds to TRAF3, thus functions as a positive regulator of IL-17 signal transduction (62). It was reported that the suppressor of cytokine signaling 2 (SOCS2) is an E3 ligase for NDR1, and the overexpression of SOCS2 inhibits NDR1-induced TNFα-stimulated NF-κB activity (63). Nevertheless, a recent study reported that NDR2 inhibits IL-17 signaling by promoting the ubiquitination and degradation of Smurf1-mediated MEKK2. Therefore, knockdown of NDR2 enhances IL-17-induced MAPK and NF-κB activation and significantly increases IL-17-induced expression of IL-6, CXCL2, and CCL20. These results suggest that NDR2 alleviates IL-17-associated inflammation (64). In conclusion, NDR1 promotes IL-17- and TNF-α-mediated inflammation while NDR2 suppresses IL-17-associated inflammation (Figure 2). Due to the crucial roles of IL-17 and TNF-α in autoimmune diseases and the contribution of NDR1 in IL-17 signaling, NDR1 could be a potential target for drug discovery of autoimmune diseases like EAE, RA, IBD, MS, and UC.

## Discussion

Taken together, we summarized the role of NDR1/2 in innate immunity by elucidating their roles in inflammation and antimicrobial immune response. Although the important roles of NDR1/2 in innate immunity have been revealed, the precise mechanism by which they regulate innate immunity are not fully illuminated. Besides that, NDR1/2 have been found to phosphorylate YAP and promote the degradation of YAP. It is reported that YAP antagonizes the antiviral innate immune response by directly binding to interferon regulatory factor 3 (IRF3) or TANK binding kinase 1 (TBK1) (65, 66). In the context of viral infection, whether NDR1/2 inhibit the production of type I interferon through YAP to enhance the antiviral immune response remains unclear. YAP impairs M2 macrophage polarization and promotes M1 macrophage activation (67). It remains unclear whether NDR1 regulates CpG-induced inflammation through modulating YAP phosphorylation and degradation. Furthermore, given the role of NDR1 in the expression and activation of STAT1 and YAP, it might be worth investigating whether NDR1 regulates macrophage polarization via phosphorylating YAP. NDR1 ablated mice are known to be more likely to develop T cell lymphoma (68). A recent study reported that NDR2 facilitates TCR-induced LFA-1 activation in T cells (69). It is intriguing to investigate the role of NDR1/2 in the adaptive immune response. Finally, both *in vitro* and *in vivo* experiments showed that NDR1/2 regulate inflammation and immune response. However, further investigation is required to ascertain the participation of NDR1/2 in human inflammation and immune response and if they could be used as therapeutic targets for immune-related diseases.

## Author Contributions

XY, HA, and YZ designed and reviewed the paper and contributed in drafting the manuscript. NO edited and reviewed the manuscript. All the authors approved the final version of the manuscript.

### Conflict of Interest

The authors declare that the research was conducted in the absence of any commercial or financial relationships that could be construed as a potential conflict of interest.
